# Sonographic Appearance of Primary Thyroid Lymphoma-Preliminary Experience

**DOI:** 10.1371/journal.pone.0114080

**Published:** 2014-12-04

**Authors:** Yu Xia, Liang Wang, Yuxin Jiang, Qing Dai, Xiaoyi Li, Wenbo Li

**Affiliations:** 1 Department of Ultrasound, Chinese Academy of Medical Sciences & Peking Union Medical College Hospital, Beijing, China; 2 Department of General Surgery, Chinese Academy of Medical Sciences & Peking Union Medical College Hospital, Beijing, China; Uppsala University, Sweden

## Abstract

**Objective:**

Primary thyroid lymphoma (PTL) is an uncommon thyroid malignancy. Despite the rarity of PTL, it is important to recognize PTL promptly because its management differs from that of all the other thyroid neoplasms. This study was designed to investigate the sonographic features of PTL.

**Methods:**

Twenty-seven pathologically confirmed PTLs were categorized into diffuse and non-diffuse type. Sonographic features including thyroid size, thyroid background echotexture, lesion size, echogenecity, calcification, vascularity, cervical lymphadenopathy of each type were retrospectively analyzed.

**Results:**

All 27 PTLs were diffuse large B-cell lymphomas and were accompanied by diffuse Hashimoto's thyroiditis. Ten were diffuse type and seventeen were non-diffuse type sonographically. The observations in diffuse group included goiter (10/10, 100.0%), marked echogenesity (10/10, 100.0%), heterogeneous echotexture (10/10, 100.0%), and cervical lymphadenopathy (4/10, 40.0%). The observations in non-diffuse group included marked hypoechogenicity (17/17, 100.0%), heterogeneous background thyroid gland (17/17, 100.0%), goiter (15/17, 88.2%), increased vascularity (8/13, 61.5%), mulifocality (10/17, 58.8%), and cervical lymphadenopathy (7/17, 41.2%).

**Conclusions:**

Although some common features were found, the sonographic appearance of PTL is unspecific, especially for the diffuse type. Therefore, interventional diagnostic procedures should be warranted in the clinical settings when PTL is suspected.

## Introduction

Primary thyroid lymphoma (PTL) is defined as a lymphoma that involves either the thyroid gland alone or the thyroid gland and adjacent neck lymph nodes without contiguous spread or distant metastases at diagnosis [1]. PTL accounts for 2.2-5% of all malignant thyroid tumors [2], [3]. Despite the rarity of PTL, it is important to recognize PTL promptly because its management differs from that of all the other thyroid neoplasms. Moreover, PTL is often curable without extensive surgery if it is diagnosed early and treated appropriately [4], [5]. Diagnosis may be established by modern imaging modalities (mainly sonography) or biopsy. Adjunctive techniques (e.g., immunohistochemical staining and flow cytometry) further increase the diagnostic accuracy. Therefore, a suspicion of PTL by the radiologist, cytologist, or clinician is important for the early diagnosis and prompt treatment of this potentially curable thyroid malignancy. The present study focused on the diagnostic performance of sonography for PTL.

## Materials and Methods

This study was approved by Peking Union Medical College Hospital ethics committee, and the ethics committee waived the need for written informed consent from the participants. And all the records data were de-identified and analyzed anonymously.

### Study population

Between May 1995 and May 2010, 27 PTLs were diagnosed in our hospital. Among the 27 PTLs, 24 were confirmed by surgical resection, and three were confirmed by ultrasound-guided biopsy. All of these PTLs displayed diffuse large B-cell lymphoma (DLBCL) pathology. The presence of Hashimoto's thyroiditis was also pathologically confirmed in all of the patients.

### US examination

Thyroid sonography was performed by using one of three scanners (GE logic 9, Philips HDI 5000, Philips IU 22) with a 5–12 MHz linear transducer. Thyroid preset was applied. All patients were scanned in a supine position with neck hyperextension. Sonograms of thyroid including cervical lymph nodes were obtained in transverse, longitudinal, and multiple oblique planes. According to the preliminary impressions of original sonography report of the PTL patient, the PTL sonography appearances in present study were divided into either the diffuse type or the non-diffuse type. In cases of diffuse type, bilateral thyroid gland was diffusely involved by neoplastic tissue without any discrete lesion (PTL or non-PTL) that was sonographically distinguishable from the adjacent parenchyma. In cases of non-diffuse type, neoplastic tissue focally involved the thyroid gland, in which one or multiple discrete lesion(s) nodularly or patchily present in thyroid. Sonographic features including thyroid size, thyroid background echotexture, lesion size, echogenecity, calcification, vascularity, cervical lymphadenopathy of each type were retrospectively determined by two radiologists (Y.X and YX.J) in consensus manner. Each of the radiologists had over 1500 cases thyroid sonography experience.

Goiter was determined as the presentation of a thyroid with the craniocaudal diameter more than 6.0 cm or the anteroposterior diameter more than 2.0 cm. The echotexture of background thyroid gland was determined as homogeneous or heterogeneous. Lesion size was analyzed as the greatest diameter of each nodule. Echogenecity that were lower than the degree of the neck strap muscle, that were between the muscle and the thyroid gland, and that were higher or equivalent to the thyroid gland were determined as markedly hypoechoic, hypoechoic, and hyperechoic or isoechoic, respectively. Calcification information such as microcalcification and macrocalcification was recorded. Dense hyperechoic with posterior shadowing was defined as macrocalcification, while tiny dot-like hyperechoic without posterior shadowing was defined as microcalcification. Compared with adjacent non-lymphomatous tissue, the vascularity was classified as avascularity (no blood flow), normal vascularity (similar to adjacent tissue), or increased vascularity (more than adjacent tissue). Lymphadenopathy was identified as lymph nodes presenting with measurements of 5 mm or greater in the short axis, and the absence of a hyperechoic hilum, a heterogeneous echotexture, chaotic vascularity, calcification, or a cystic change. In the cases of diffuse type, vasucarity were not evaluated because no non-lymphomatous tissue was available for comparison.

### Statistical analysis

Statistical analyses were performed with SPSS 13.0 software package (SPSS, Chicago, IL). Only descriptive analysis was used in this study. Continuous variables were summarized as means ± SD and categorical variables as percentages.

## Results

### Clinical data

Among twenty seven patients, 17 patients were female, 10 patients were male. The patients were between 25 and 85 years old, and the average age was 64 years. Sixteen (16/27, 59.2%) PTL patients presented with rapid thyroid enlargement over the prior 2 months. Six (6/27, 22.2%) patients had breathing difficulty. Ten patients (10/27, 37%) showed subclinical hypothyroidism, the other patients showed euthyroidism. Of the 21 patients who had thyroid antibody test results, 17 (17/21, 81%) displayed elevated levels of thyroid antibodies, such as anti-TG or anti-TPO antibodies.

### Sonographic appearance of diffuse-type PTL

**Figure 1:**
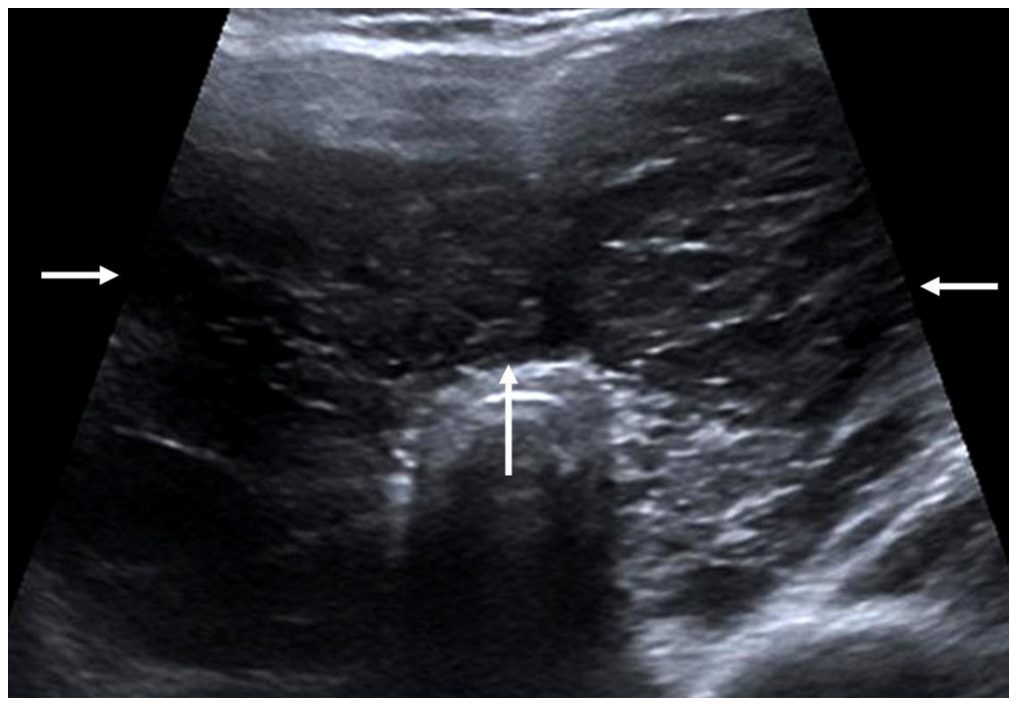
A 50-year-old female patient with diffuse PTL. Transverse sonogram shows the enlarged thyroid with decreased heterogeneous internal echoes (arrows).

**Figure 2:**
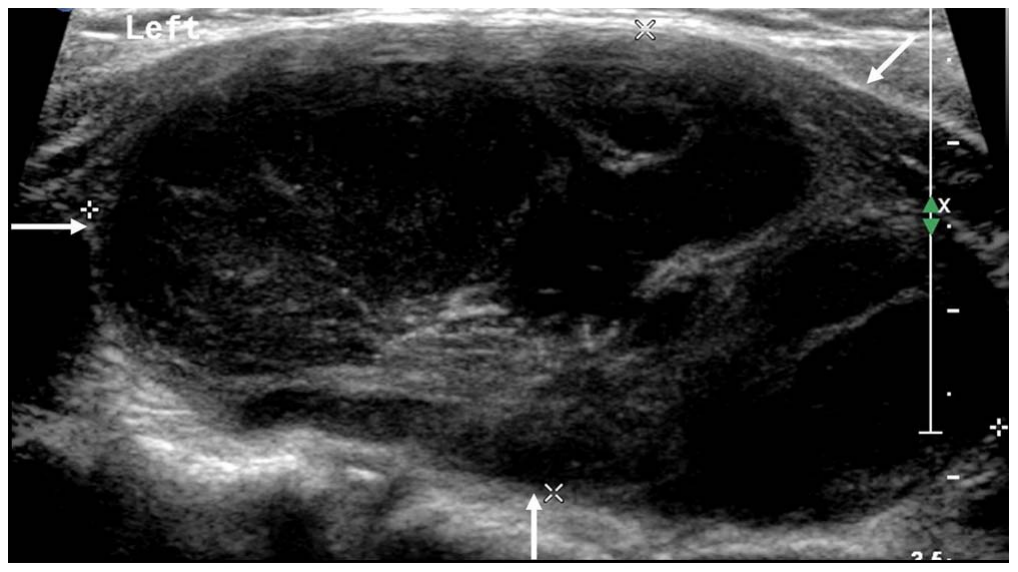
A 71-year-old female patient with diffuse PTL. The longitudinal sonogram of left lobe shows the marked hypoechogenecity with posterior acoustic enhancement (arrows).

**Figure 3:**
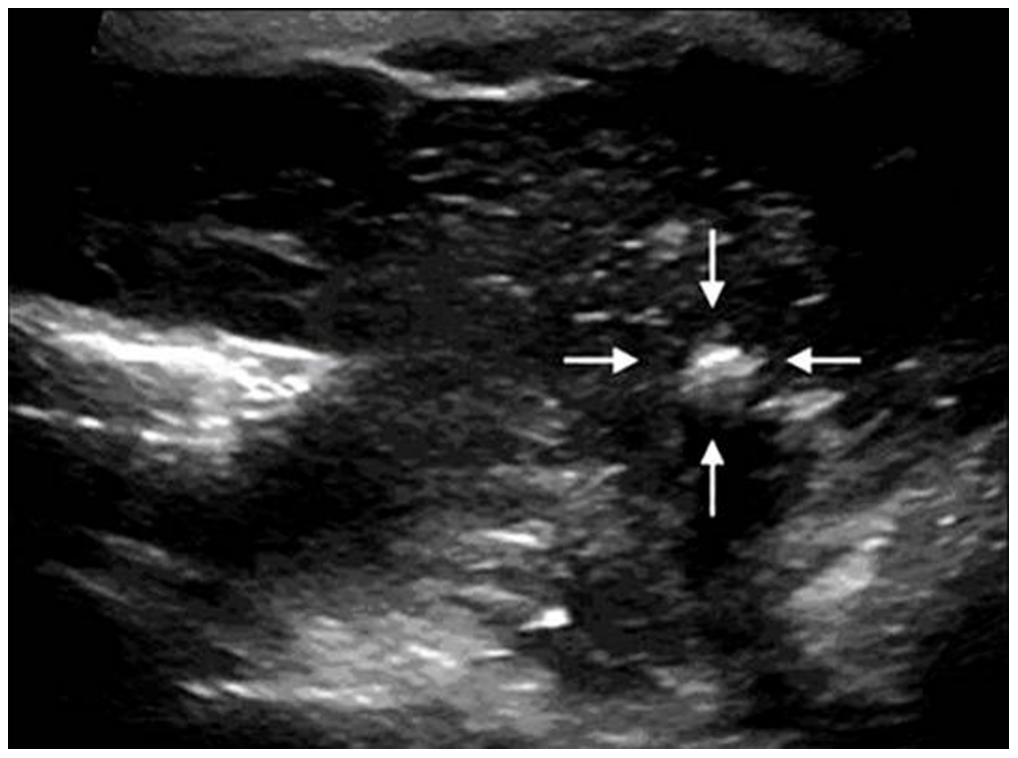
A 55-year-old male patient with diffuse PTL. The transverse sonogram shows the presence of macrocalcification (arrows).

Ten (10/27, 37.0%) diffuse PTLs were included in the present study. The sonographic features of these PTLs are summarized in Table 1. All 10 of the diffuse PTLs had goiter, heterogeneous echotexture and marked hypoechogenecity with posterior acoustic enhancement (Figure 1,2). Macrocalcification was present in one case (Figure 3), cervical lymphadenopathies were presented in four patients.

**Table 1:** Sonographic appearance of diffuse PTL.

Case No.	Gender	Age	Goiter	Echogenecity	Echotexture	Calcification	Cervical lymphadenopathy
1	Male	65	+	Markedly hypoechogenecity	Heterogeneous	−	+
2	Male	71	+	Markedly hypoechogenecity	Heterogeneous	−	−
3	Female	71	+	Markedly hypoechogenecity	Heterogeneous	−	−
4	Female	48	+	Markedly hypoechogenecity	Heterogeneous	−	+
5	Female	54	+	Markedly hypoechogenecity	Heterogeneous	−	−
6	Male	48	+	Markedly hypoechogenecity	Heterogeneous	−	+
7	Female	61	+	Markedly hypoechogenecity	Heterogeneous	−	−
8	Male	55	+	Echogenecity	Heterogeneous	+ (macrocalcification)	−
9	Female	50	+	Markedly hypoechogenecity	Heterogeneous	−	+
10	Female	47	+	Markedly hypoechogenecity	Heterogeneous	−	−

### Sonographic appearance of non-diffuse-type PTL

**Figure 4:**
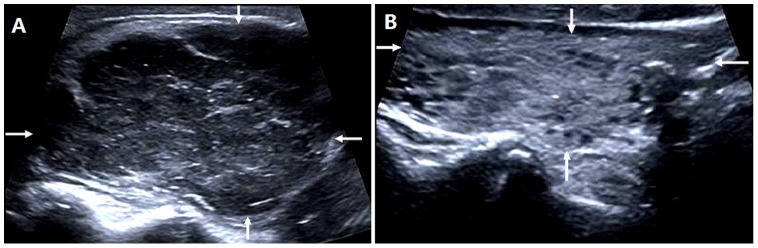
A 67-year-old male patient with non-diffuse PTL. (A)The longitudinal sonogram shows that PTL was limited to the right thyroid lobe (arrows). (B) The longitudinal sonogram shows a heterogeneous echotexture of the left thyroid lobe (arrows).

**Figure 5:**
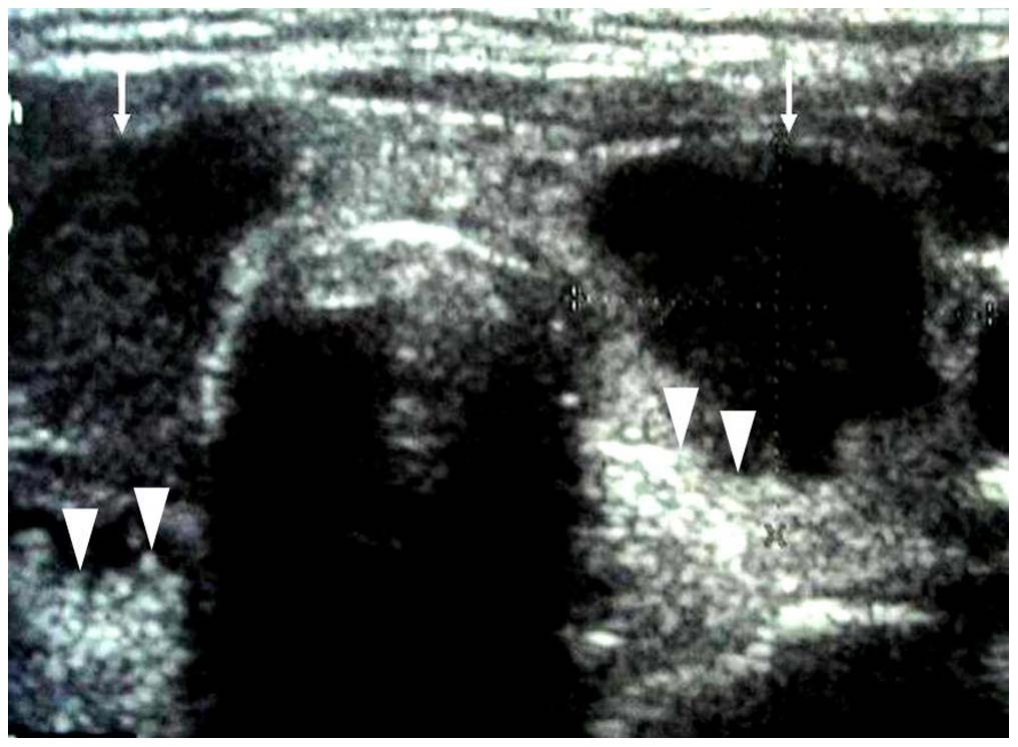
A 78-year-old female patient with non-diffuse PTL. The transverse sonogram shows the extremely hypoechoic lesions (arrows) with posterior acoustic enhancement (arrow heads).

**Figure 6:**
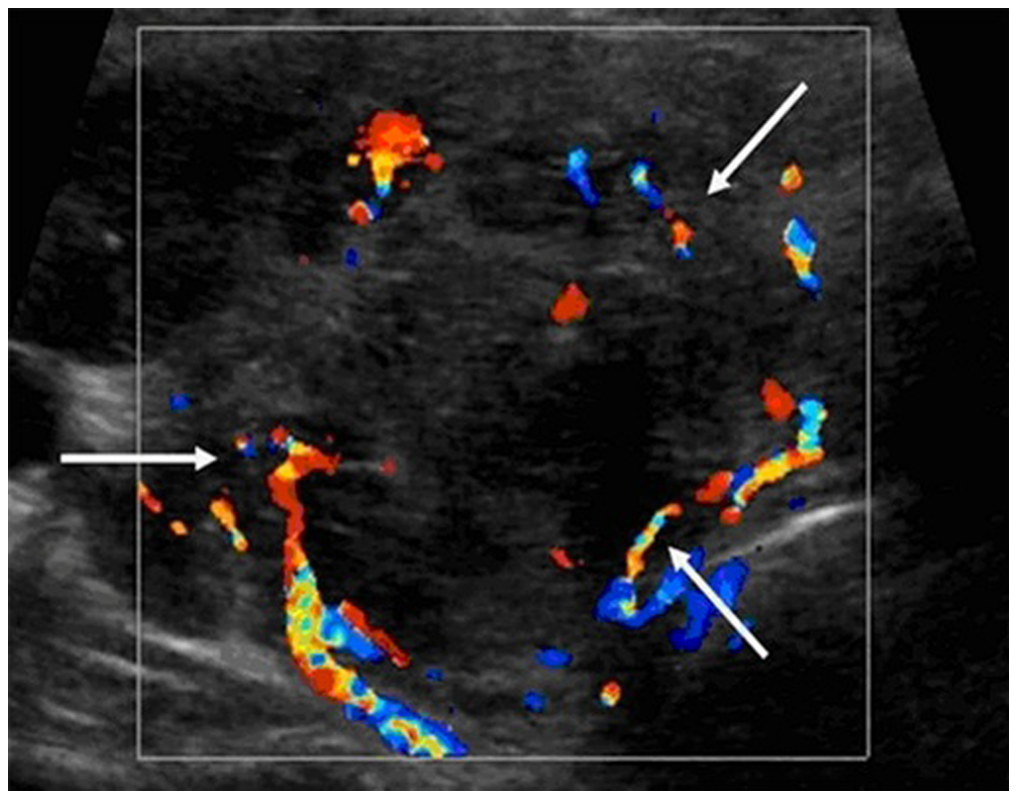
A 56-year-old female patient with non-diffuse PTL. The transverse sonogram shows the increased chaotic vascularity (arrows). (Tr: Trachea)

**Figure 7:**
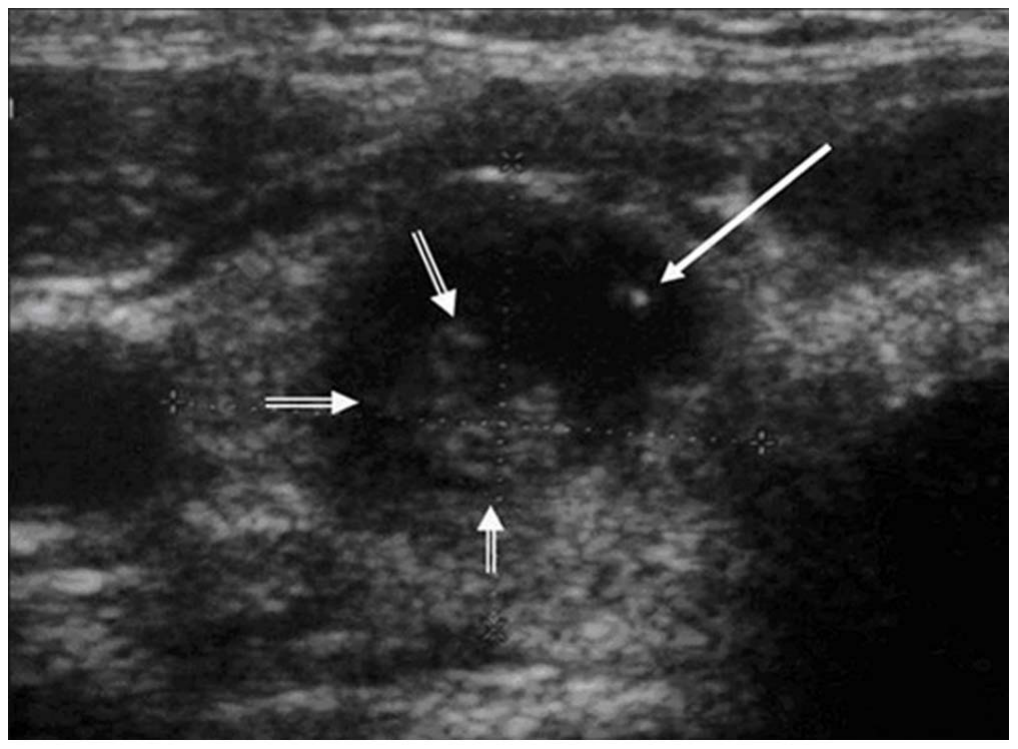
A 78-year-old female patient with non-diffuse PTL. The transverse sonogram shows the presence of a hyperechoic portion (short arrows) and microcalcification (long arrow) within an extremely hypoechoic PTL lesion.

**Figure 8:**
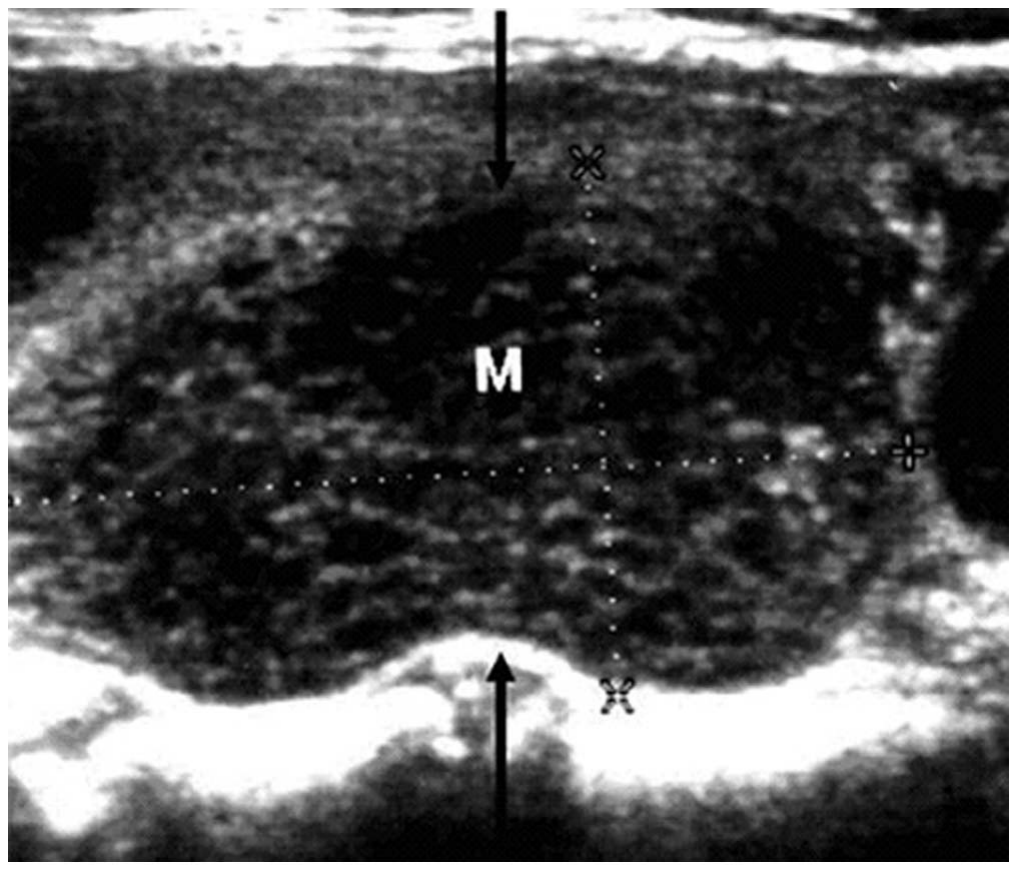
A 65-year-old female patient with non-diffuse PTL. The longitudinal sonogram shows the honeycomb appearance in a PTL lesion (arrows). (M: mass)

The present study included 17 (17/27, 63.0%) non-diffuse PTLs, and the sonographic features of these PTLs are summarized in Table 2. Most of the patients with non-diffuse PTLs presented with goiter (15/17, 88.2%), and all of the non-diffuse PTL patients displayed heterogeneous echotexture of the thyroid gland (17/17, 100.0%) (Figure 4B). All of the non-diffuse PTLs were markedly hypoechoic with posterior acoustic enhancement (Figure 4A, 5). In most cases, PTLs showed mulifocality (10/17, 58.8%) and increased vascularity (8/13, 61.5%) (Figure 6). Microcalcification was identified in one case (Figure 7). Interestingly, a honeycomb appearance, which was due to the presence of reticular echogenic strands, was present in one case (Figure 8). Seven (7/17, 41.2%) patients presented cervical lymphadenopathies.

**Table 2:** Sonographic appearance of non-diffuse PTL.

Features	No.(%)
Size (cm)	3.1±1.6 cm
Number	
Solitary	7/17 (41.2%)
Multiple	10/17 (58.8%)
Echogenecity	
Markedly hypoechoic	17/17 (100.0%)
Hyper-/iso-/hypoechoic	0/17 (0.0%)
Calcification	
Microcalcification	1/17 (5.9%)
Macrocalcification	0/17 (0.0%)
None	16/17 (94.1%)
Vascularity	
Avascularity	2/13(15.4%)
Normal vascularity	3/13(23.1%)
Increased vascularity	8/13 (61.5%)
Background thyroid gland	
Goiter	15/17 (88.2%)
Heterogeneous echotexture	17/17 (100.0%)
Cervical lymphadenopathy	7/17 (41.2%)

## Discussion

PTL is an uncommon pathology and constitutes less than 5% of all thyroid malignancies. It is more common in females than in males; most PTL patients are diagnosed in their 6^th^ or 7^th^ decade of life. However, for the reason of its therapeutic strategies is fundamentally distinct from other thyroid malignancies, this rare disease deserves more attention. Currently, PTL therapy includes local therapy alone (surgery or radiotherapy) or, most commonly, combined multimodality treatment (mainly chemoradiation therapy). The prognosis of PTL patients depends on the histologic grade of the tumor and the stage of the disease. Intrathyroidal disease 5-year survival is 90% and decreases to 35% for patients with extrathyroid disease. Overall, 5-year survival ranges between 50 and 60% [6].

Preexisting Hashimoto's thyroiditis is a well-recognized risk factor for the development of PTL. The incidence of Hashimoto's thyroiditis in patients with PTL has been reported to be between 30–100% in previous studies (the average incidence was 80%) [7]. In present study, Hashimoto's thyroiditis background disease was pathologically confirmed in all of the patients.

Ota described the sonographic appearance of PTL in the largest population currently reported in the literature, and he classified PTL into nodular, diffuse, and mixed types based on the sonographic analysis.^8^ In Ota's study, the positive predictive value of US in nodular and mixed type was similar and significantly higher (64.9% and 63.2%) than that in diffuse type (33.7%). On the other hand, in routine thyroid sonography practice, either diffuse disease or non-diffuse diffuse disease is mostly described in reports. Therefore, we proposed a new classification by combining the nodular and mixed types into the non-diffuse type.

All diffuse PTLs that were examined in the present study showed goiter, markedly hypoechoic and heterogeneous internal echoes. These observations are similar to the characteristic appearance of diffuse Hashimoto's thyroiditis. Posterior acoustic enhancement was reported to be useful in discriminating PTL from severe Hashimoto's thyroiditis [8]. However, we noticed that this feature could also be observed in some diffuse Hashimoto's thyroiditis cases. Therefore, agreeing with Podoloff's [9] opinion, we thought there was no specific sonographic feature for diffuse PTL. But it doesn't mean there is no any clue for screening this rare disease. Rapid growth (usually within the previous 1-3 months), painless thyroid enlargement, and pressure symptoms (mainly involving the aerodigestive tract) are the common clinical presentations of PTL [10]. These variables, however, are not commonly present in diffuse Hashimoto's thyroiditis. In the present study, these clinical features were observed in most diffuse PTL patients (7/10, 70.0%). Furthermore, in the relevant clinical settings, sonography can also effectively narrow down the differential diagnosis of PTL from other thyroid diseases, such as undifferentiated thyroid carcinoma, hemorrhage into an adenoma or cyst, and subacute thyroiditis [11], [12]. Therefore, the combination of sonographic and clinical features may facilitate the diagnosis of diffuse PTL.

Compared to diffuse type, the recognition of non-diffuse type was relatively easy on sonography due to its mass effect. Several common sonographic features were noticed in non-diffuse PTLs. The constellation of these findings may aid in the diagnosis of non-diffuse PTL. However, the present study lacked a single sonographic feature that could offer us a confident diagnosis of non-diffuse PTL.

Goiters were frequently observed in non-diffuse PTLs in the present study (88.2%). This may be partially due to the larger size (3.1 cm in greatest diameter) and rapid growth of the PTL lesion. Meanwhile, multiple occurrences of PTL lesions may also contribute to the development of goiter. Most non-diffuse PTLs (65.0%) presented multiple lesions in current study. Goiter and rapid progression is also observed in undifferentiated thyroid carcinoma. The differentiation of PTL from undifferentiated thyroid carcinoma is important because the management and prognosis of these two diseases is very different. Undifferentiated thyroid carcinoma often presents calcifications and cystic changes that can be observed on sonograms [13]. In the present study, however, only one non-diffuse PTL (5.9%, 1/17) presented calcification.

Marked hypoechogenicity is the most typical appearance of non-diffuse PTL [14]. All 17 non-diffuse PTLs included in the present study were markedly hypoechoic. Due to the extremely low level of internal echoes, nodular PTLs have been described as “pseudocysts” in previous studies [14]. According to the management guidelines for thyroid nodules [15], these thyroid lesions should all be considered for suspicious malignancy.

Calcification in PTL is rare. Microcalcification presented in one case of non-diffuse PTL, while macrocalcification presented in one case of diffuse PTL. The relatively low incidence rate of calcification may be useful in choosing an appropriate method of biopsy. Usually, for papillary thyroid carcinoma, FNA was more commonly used. However, for PTL, core biopsy was reported to be a more productive manner [16].

Increased chaotic vascularity in the thyroid nodules is a helpful sonographic finding for the screening of thyroid malignancy and prompt FNA or core biopsy [15]. In current study, increased chaotic vascularity was observed in 8 non-diffuse PTLs (61.5%, 8/13). However, it remained unclear whether this hypervascularity was associated with the large size of the PTLs. Given the limited number of patients in the present study, we were unable to provide a conclusive determination. Conclusions should be drawn from further large-sample investigations with size-stratified analyses.

In conclusion, sonography alone is quite likely to miss diffuse type PTL due to the similar sonographic appearance between diffuse PTL and severe diffuse Hashimoto's thyroiditis. However, some clinical presentations, such as rapid thyroid enlargement and breathing difficulty, may be helpful in the diagnosis of diffuse PTL. Markedly hypoechoic, goiter, multifocal, hypervascular, absent of calcification, and showed goiter were features of non-diffuse type PTL. Therefore, thyroid lesions with these sonographic and clinical features should warrant further investigation, e.g. core needle biopsy, to rule out the possibility of non-diffuse PTL.
